# Demographics of the injury pattern in severely injured patients with an associated clavicle fracture: a retrospective observational cohort study

**DOI:** 10.1186/1749-7922-8-36

**Published:** 2013-09-22

**Authors:** Jacqueline JEM van Laarhoven, Steven Ferree, R Marijn Houwert, Falco Hietbrink, EgbertJan MM Verleisdonk, Luke PH Leenen

**Affiliations:** 1 Department of Surgery, University Medical Center Utrecht, Heidelberglaan 100, Utrecht, CX, 3584, The Netherlands; 2 Department of Surgery, Diakonessenhuis Utrecht, Bosboomstraat 1, Utrecht, KE, 3582, The Netherlands

**Keywords:** Clavicle fracture, Trauma care, ISS, Severely injured, Associated injury pattern

## Abstract

**Background:**

Despite an increasing interest in the treatment of clavicle fractures, this is still a not yet defined area in severely injured patients as most studies exclude these patients. Analyzing fracture type and evaluate accompanying injuries can provide valuable information in an early stage of trauma care.

**Objective:**

To identify prevalence, fracture type and accompanying injuries of clavicle fractures in the severely injured patient.

**Methods:**

We included all severely injured patients (ISS ≥ 16) with a clavicle fracture from January 2007 - December 2011. We prospectively collected data about demographics, injuries, trauma mechanism and mortality. Fractures were classified using the Robinson classification.

**Results:**

A total of 1534 patients had an ISS ≥16, of which 164 (10.7%) patients had a clavicle fracture. Traffic related accidents were the main cause of injury (65%). Most fractures were midshaft fractures (66.5%) of which 56% were displaced. Seven patients were treated operatively. There was no significant difference in ISS between the three fracture types. 83% of the patients sustained additional injury to the head and neck; the most prevalent injuries were skull or skull base fractures (41.5%) and maxillofacial fractures (29%). Furthermore 77% of the patients had additional thoracic injury; the most prevalent injuries were rib fractures (59%) and a pneumothorax (38%). The mortality rate was 21.4%.

**Conclusion:**

A clavicle fracture was present in more than 10% of the severely injured patients. Displaced midshaft clavicle fractures were the most common type of fracture. Additional injuries to the head and neck region occurred in 83% of the patients and thoracic injuries occurred in 77% of the patients.

## Introduction

Clavicle fractures account for approximately 5% of all fractures. Most often it concerns a midshaft clavicle fracture (80%) of which 50% is dislocated [1,2]. In the past years there has been increasing interest in the treatment of clavicle fractures, especially in the midshaft fractures. However, most studies evaluating treatment of clavicle fractures exclude severely injured trauma patients [3,4]. Therefore the clavicle fracture in the severely injured patient is a not yet defined area.

Advanced Trauma Life Support (ATLS) principles advocate that in all severely injured trauma patients a chest x-ray is made to identify potential thoracic injuries [5]. Treatment-dictating injuries are frequently missed at the chest x-ray as 50% of all rib fractures and a significant number of hemato- and pneumothorax are not identified [6,7]. Clavicle fractures, on the other hand, can almost always be diagnosed at chest x-ray. Therefore it is of great interest to analyze which accompanying injuries most frequently occur in severely injured patients with a clavicle fracture. These “expected” associated injuries can be taken into account in an early stage of trauma care for severely injured patients.

The aim of this study is to identify prevalence, fracture type and accompanying injuries of clavicle fractures in the severely injured patient.

## Materials and methods

Patients included in this study were those admitted in a level 1 trauma center from January 2007 until December 2011. The organisation of trauma care in the Netherlands is based on the American model of trauma regionalization. The Netherlands is divided in 11 separate trauma regions, each region contains a level one trauma center [8].

In this study prospective data from the Dutch National Trauma Database (DNTD) for the area Central Netherlands were used. The DNTD contains documentation on all trauma patients that are treated at the emergency department and subsequently admitted. Data in the DNTD were collected in a standardized manner and include detailed information on demographics, trauma event and mechanism, primary trauma survey, initial treatment and injuries. Injuries were diagnosed at primary survey, subsequent surgery or during admission. Thoracic and pelvic x-ray imaging were performed for all trauma patients and when indicated supplemented with ultrasound and computed tomography (CT). The database accuracy is constantly evaluated by two database managers.

All injuries were coded using Abbreviated Injury Scale (AIS) location codes allocated to one of the six body regions (head and neck, face, chest, abdomen, extremities and external) to calculate the Injury of Severity Score (ISS) [9]. Patients with a clavicle fracture were selected using AIS location codes. The ISS provides an overall score for patients with multiple injuries and is used to determine injury severity; 0 corresponds with no injury, the maximum score of 75 corresponds with injury leading to death [10]. Patients with an ISS ≥ 16, obtained from ≥2 AIS regions and physiological alterations due to the injuries are considered severely injured and were included in our analysis [11].

For these patients, age, gender, trauma mechanism, injured side, additional injuries, department of admission (Intensive care Unit, Medium Care Unit, Operation Room) and discharge facility were collected from the DNTD. In all patients trauma mechanism was analysed and determined if it was a high energy trauma. The ATLS definition for high energy trauma was used [5]. Furthermore death associated with the trauma was obtained from the electronic patient documentation (EPD).

To evaluate the clavicle fractures we used the imaging studies performed. These radiological tests allowed for clear images of the fracture and of possible dislocation in anterior-posterior or cranial-caudal direction. Fractures were classified by the researchers (JL, SF and MH) using the Robinson classification. This classification divides the clavicle in a medial fifth (type 1), a diaphyseal part (type 2) and a lateral fifth (type 3). This is further divided by three other variables; intra-articular extent, degree of comminution, and degree of displacement [12].

### Data analysis

Mean numbers were noted with standard deviation (SD), median numbers were noted with interquartile range (IQR). Statistical analysis was performed using the *χ*^2^ test for categorical variables and *t*-test and one-way-ANOVA for continuous variables. Binary logistic regression was used for the calculation of the dependent variables in additional injuries. A p-value of ≤0.05 was considered significant. Data were analyzed with SPSS Version 20.0, Chicago, IL, USA.

## Results

**Figure 1:**
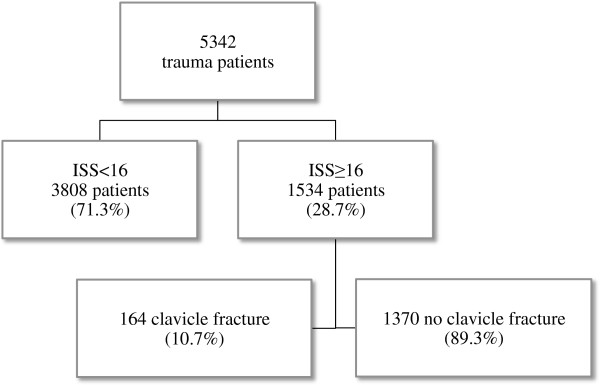
Flowchart selection of the studied population of trauma patients at the University Medical Center Utrecht from 2007 until 2012.

A total of 5357 trauma patients were treated at the emergency department and subsequently admitted over the 5 year period (January 2007- December 2011). Of these patients 1534 had an ISS of 16 or higher, of which 164 (10.7%) patients had a clavicle fracture (Figure 1). The mean age of the entire studied population was 45.8 (± 21.9), four patients were aged under 16 years and 160 patients were aged 16 years and older (Table 1). Patients were predominantly male (66.5%). The main part of patients (65%) were involved in traffic accidents and 112 patients sustained a high energy trauma. The mortality rate was 21.4%. The majority of patients died due to injury to the central nervous system (74.3%), other causes were organ failure (14.3%), exsanguinations (8.6%) and one patient died due to sepsis. There were no missing data in baseline characteristics (Table 1). Most of the fractures were midshaft clavicle fractures (66.5%) of which 56% were displaced (Table 2).

**Table 1:** Demographics of the studied population of severely injured patients with a clavicle fracture

			**Clavicle fracture**
**Age overall**			45.8 (± 21.9)
**Age**	**Type I**		39.1 (± 22.7)
	**Type II**		44.0 (± 20.8)
	**Type III**		56.0 (± 20.4)
**Gender (M/F)**			110/54
**Trauma mechanism**	**Traffic**	**Car**	34 (20.7%)
		**Motor**	36 (22.0%)
		**Bike**	32 (19.5%)
		**Pedestrian**	6 (3.7%)
	**Sports**		1 (0.6%)
	**Fall**		47 (28.6%)
	**Other**		8 (4.9%)
**Injured side (L/R/both)**			92/70/2
HET*			115 (70.1%)
ISS **			29.4 (± 10.4)
**Admission at Intensive Care Unit**			64 (39.0%)
**Admission at Medium Care Unit**			40 (24.4%)
**Direct transport to OR**			22 (13.4%)
**Mortality**	**At emergency room**		2 (1.2%)
	**Within < 24 hours**		17 (10.4%)
	**During admission**		16 (9.8%)

*Patients involved in an high energy trauma ** Injury of Severity Score.

**Table 2:** Robinson classification of clavicle fractures in severely injured patients

**Robinson classification**	**No. of patients (% of population)**	**Mean age ± SD**	Mean ISS* ± SD
1A	8 (4.9%)	33.9 (± 20.6)	36.3 (± 11.2)
1B	2 (1.2%)	60.0 (± 24.0)	27.5 (± 9.1)
2A	51 (31.1%)	48.9 (± 22.7)	29.2 (± 9.5)
2B	61 (37.2%)	39.5 (± 18.3)	29.8 (± 11.8)
3A	32 (19.5%)	57.5 (± 21.0)	29.0 (± 9.7)
3B	10 (6.1%)	51.3 (± 18.3)	23.7 (± 4.8)

*Injury of Severity Score.

Patients with type III fractures were older than patients with type I (P = 0.022; 16.9 95% CI 2.43-31.37) or II fractures (P = 0.001; 12.2 95% CI 4.78-19.65). No difference in age was found between patients with type I and II fractures. Patients with a displaced fracture are significantly younger than patients with a non-displaced fracture (P = 0.006; 8.933, 95% CI 2.5-15.3). There was no significant difference in ISS between the three groups and no significant difference in ISS in patients with a displaced or non-displaced fracture.

In 7 patients, the clavicle fracture was treated operatively, the mean time was admission day 5 (range 1-11 days). All patients received plate fixation. In one case it concerned a type 1B fracture, in 5 cases a type 2B fracture and in one case a type 3B fracture. One patient was directly transferred and the remaining 153 patients were treated conservatively (Table 3).

**Table 3:** Treatment of clavicle fractures in severely injured patients treated at the University Medical Center Utrecht, classified by the Robinson classification

**Robinson classification**	**Operative**	**Conservative**
1A	0	8
1B	1	1
2A	0	50
2B	5	54
3A	0	32
3B	1	9
**Total**	**7**	**154**

**Figure 2:**
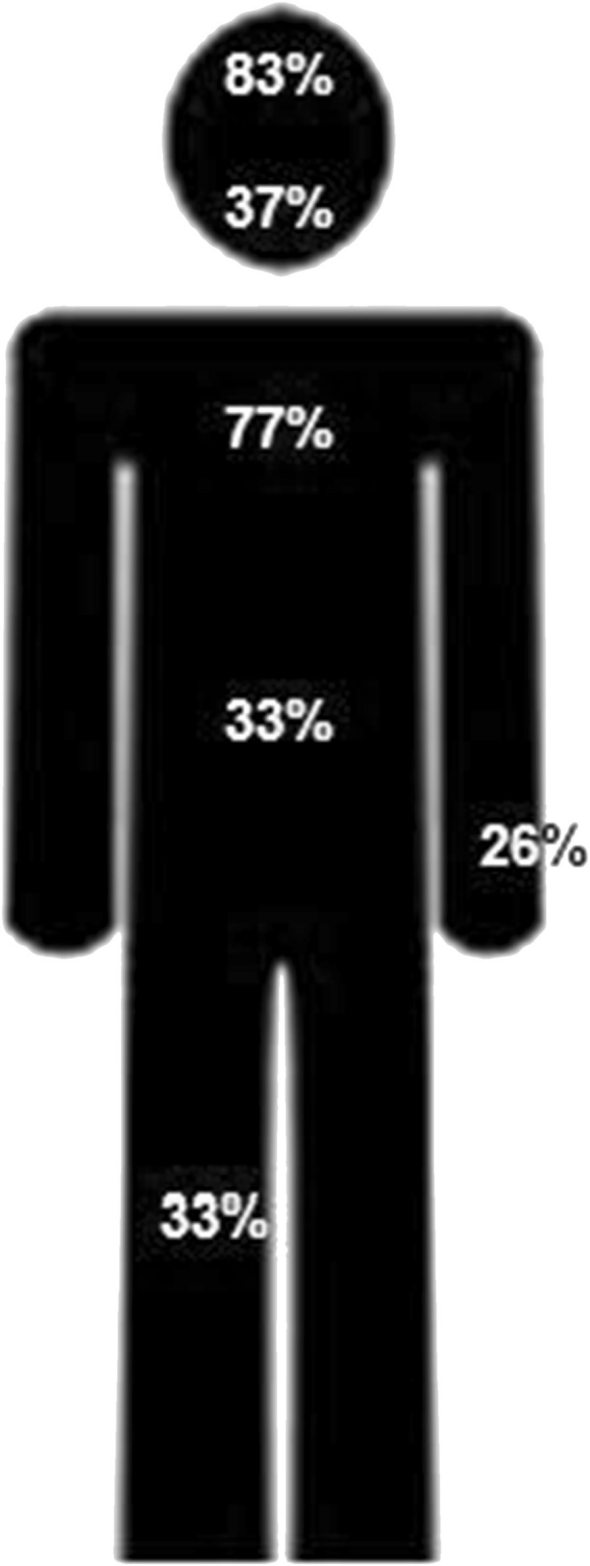
Additional injuries in severely injured patients with a clavicle fracture.

Of all patients, 83% sustained additional injuries to head and neck. The most prevalent injury was a skull or skull base fracture (41.5%) followed by maxillofacial fractures in 29%. Seventy-seven percent had additional thoracic injuries (Table 4; Figure 2), 59% of the patients had rib fractures and 38% of the patients had a pneumothorax. There was no significant difference in displaced and undisplaced fractures concerning additional injuries.

**Table 4:** Additional injuries in severely injured patients per type of clavicle fracture

	**Upper extremity**	**Lower extremity**	**Abdominal injury**	**Thorax injury**	**Face injury**	**Head & neck injury**
	**n (%)**	**n (%)**	**n (%)**	**n (%)**	**n (%)**	**n (%)**
**Type I fracture (n = 10)**	3 (30.0 %)	4 (40.0%)	4 (40.0%)	9 (90.0%)	1 (10.0%)	6 (60.0%)
**Type II fracture (n = 112)**	33 (29.7%)	36 (32.4%)	38 (34.2%)	88 (79.3%)	43 (38.7%)	90 (82.6%)
**Type III fracture (n = 42)**	7 (16.7%)	13 (31.0%)	11 (26.2%)	28 (66.7%)	16 (38.1%)	37 (88.1%)
**No of patients (% of population)**	43 (26.4 %)	53 (32.5%)	53 (32.5%)	125 (76.7%)	60 (36.8%)	133 (82.6%)

## Discussion

The main findings of this study were that 10% of all severely injured patients had a clavicle fracture and 21.4% of multitrauma patients with a clavicle fracture died during trauma care or admission. Midshaft clavicle fractures were most common and 44% of all fractures were displaced. Eighty-three percent of our patients had additional head and neck injuries and 77% had additional thoracic injuries.

Two large epidemiologic studies report incidence rates of clavicle fractures in the normal population between 2,6 and 4% [1,2]. Therefore clavicle fractures seem to occur at least twice as common in severely injured patients. In comparison to the study of Robinson et al, less fractures in our population were displaced. This difference might be explained by the fact that in severely injured patients, energy forces are distributed over the body. This is different compared to the direct energy on the clavicle in case of a single fracture [13,14]. Results of this study indicate that the clavicle is the gate-keeper of the thorax in severely injured patients. This hypothesis can be supported by the high rate of additional thoracic injuries.

The overall mortality of the study population was 21.4%, which includes deaths at the emergency room. Our results are similar to an abstract published by Mc Kee et al, which showed that in multitrauma patients the presence of a clavicle fracture was found to be associated with a mortality rate of 32% (thirty-four of 105 patients), mainly due to concomitant chest and head injuries [15].

Previous studies have been performed to identify associated injury in patients with upper extremity injury. Analysis showed significantly more rib fractures (52.9%), lung injuries (47.1%) and spinal fractures (29.1%) in patients with scapula fractures [16]. Also a correlation between shoulder girdle injuries and rates of head (31.5%), great vessel (3.9%) and thoracic injury (36.8%) has been described [17]. Compared to scapula and upper extremity injury a clavicle fracture is more likely to be identified on chest x-ray. Therefore clavicle fractures are a good predictor for additional injury and can be better identified and used in an early stage. Horst et al. found a correlation between a clavicle fracture and additional upper extremity injuries in polytrauma patients [18]. Therefore the clavicle fracture can also play an important role in the tertiary survey.

This study represents an analysis based on a prospective database, although retrospectively analyzed, and is one the first to analyze clavicle fractures in the severely injured patients. Because of the detailed description of all injuries, we were able to perform a profound analysis.

The DNTD includes patients who were treated at the Emergency Room of our hospital and subsequently admitted. Therefore patients with a clavicle fracture and an ISS ≥ 16 who were not admitted, are not included in our database. Considering the additional injuries in case of an ISS ≥ 16 we can safely assume that the number of patients we missed is small and this database provides a representative study population.

## Conclusion

Clavicle fractures occur frequently (10%) in severely injured patients and 21,4% of the patients died during trauma care or admission. Midshaft clavicle fractures were most common and 44% of all fractures were displaced. Eighty-three percent of our patients had additional head and neck injuries and 77% had additional thoracic injuries.

## Competing interests

The authors declare that they have no competing interests.

## Authors’ contributions

All authors: 1) have made substantial contributions to conception and design, or acquisition of data, or analysis and interpretation of data; 2) have been involved in drafting the manuscript or revising it critically for important intellectual content; 3) have given final approval of the version to be published. JL: Study conception and design, acquisition of data, analysis and interpretation of data, drafting of manuscript. SF: Acquisition of data, analysis and interpretation of data, drafting of manuscript. MH: Study conception and design, analysis and interpretation of data, drafting of manuscript. FH: Study conception and design, analysis and interpretation of data, critical revision. EV: Analysis and interpretation of data, critical revision of manuscript. LL: Study conception and design, critical revision of manuscript. All authors have given final approval for this manuscript to be published.
